# Lost in the System: Responsibilisation and Burden for Women With Multiple Long‐Term Health Conditions During Pregnancy

**DOI:** 10.1111/hex.14104

**Published:** 2024-06-14

**Authors:** Stephanie J. Hanley, Sharon McCann, Siang Ing Lee, Zoe Vowles, Rachel Plachcinski, Krish Nirantharakumar, Mairead Black, Louise Locock, Beck Taylor

**Affiliations:** ^1^ Institute of Applied Health Research University of Birmingham Birmingham West Midlands UK; ^2^ Health Services Research Unit University of Aberdeen Aberdeen Aberdeenshire UK; ^3^ Department of Women and Children's Health Guy's and St. Thomas' NHS Foundation Trust London UK; ^4^ Aberdeen Centre for Women's Health Research University of Aberdeen Aberdeen Aberdeenshire UK; ^5^ Department of Health Sciences University of Warwick Warwick West Midlands UK

**Keywords:** integrated health care systems, multimorbidity, multiple long‐term conditions, obstetrics, patient centred care, qualitative research

## Abstract

**Introduction:**

Over a fifth of pregnant women are living with multiple long‐term health conditions, which is associated with increased risks of adverse outcomes for mothers and infants. While there are many examples of research exploring individuals' experiences and care pathways for pregnancy with a single health condition, evidence relating to multiple health conditions is limited. This study aimed to explore experiences and care of women with multiple long‐term health conditions around the time of pregnancy.

**Methods:**

Semistructured interviews were conducted between March 2022 and May 2023 with women with multiple long‐term health conditions who were at least 28 weeks pregnant or had had a baby in the last 2 years, and healthcare professionals with experience of caring for these women. Participants were recruited from across the United Kingdom. Data were analysed using thematic analysis.

**Results:**

Fifty‐seven women and 51 healthcare professionals participated. Five themes were identified. Women with long‐term health conditions and professionals recognised that *it takes a team* to avoid inconsistent care and communication, for example, medication management. Often, women were required to take a *care navigation* role to link up their healthcare providers. Women described mixed experiences regarding care for their *multiple identities and the whole person.* Postnatally, women and professionals recognised a downgrade in care, particularly for women's long‐term health conditions. Some professionals detailed the importance of engaging with women's knowledge, and recognising their own professional *boundaries of expertise*. Many participants described difficulties in providing *informational continuity* and subsequent impacts on care. Specifically, the setup of care systems made it difficult for everyone to access necessary information, especially when care involved multiple sites.

**Conclusion:**

Pregnant women with long‐term health conditions can experience a substantial burden of responsibility to maintain communication with their care team, often feeling vulnerable, patronised, and let down by a lack of acknowledgement of their expertise. These results will be used to inform the content of coproduction workshops aimed at developing a list of care recommendations for affected women. It will also inform future interventional studies aimed at improving outcomes for these women and their babies.

**Patient or Public Contribution:**

Our Patient and Public Involvement group were involved in the design of the study and the analysis and interpretation of the data, and a public study investigator was part of the author group.

## 1 Introduction

Over 20% of pregnant women have multiple long‐term health conditions (MLTC) [1], which is associated with an increased risk of adverse outcomes for mothers and infants [2, 3]. Most maternal deaths involve coexisting physical and/or mental health conditions [4, 5]. A clinical review of the pregnancy care of 61 UK women with MLTC identified that care was not sufficiently integrated or individualised, and staff were inexperienced in pregnancy medicine and managing complex needs, resulting in poor outcomes including stillbirth [6]. The review also identified that women may have substantial psychological needs, and may find it challenging to engage with care.

Many studies have explored experiences and care for pregnancy with a single morbidity and have shown that women feel stigmatised due to their conditions, they prioritise their child's health and needs over their own, they are often unable to access professional psychological support, especially in the postnatal period when they are struggling to reestablish control over their conditions, but they feel reassured when they are able to access specialist clinics, including for preconception and medication counselling [7, 8, 9, 10, 11, 12]. However, evidence relating to MLTC and the potential cumulative effects on women's maternity care and experiences is limited. While substantial work exists concerning MLTC experiences [13, 14, 15, 16], it is under researched within pregnancy. A Danish study of experiences of long‐term conditions during pregnancy where half of participants had MLTC reported that care focused on the chronic conditions, with reduced choice, fragmented care, superficial midwifery care, and women taking responsibility for communication between professionals [17]. A UK study of health professionals and pregnant women with MLTC identified communication and referral pathway gaps and differing professional and patient priorities [18]. In both studies women reported care being more baby than woman‐focused with a reduction in support postbirth.

The existing evidence offers valuable insights but is relatively limited. Multimorbidity is heterogeneous, ranging from mild to severe problems, few or many conditions, and combinations of physical and mental health diagnoses. Further evidence is needed to clarify and address the challenges in providing integrated, person‐centred care across different conditions and contexts, to identify good practice, and priorities for change. This study aimed understand the experiences of both women and healthcare staff to help address this gap in evidence. It is part of the wider mixed‐methods MuM‐PreDiCT consortium (mumpredict.org) working to characterise and understand the predictors, determinants, and consequences of MLTC in pregnant women.

## 2 Materials and Methods

The reporting of this study follows the consolidated criteria for reporting qualitative research (COREQ) guidelines [19].

This was a UK‐wide qualitative interview study, adopting an interpretivist approach, to understand experiences of receiving and providing maternity care of women with MLTC, and healthcare professionals. National Health Service (NHS) Health Research Authority ethical approval was granted by Wales Research Ethics Committee 7 (Lumivero [20]/WA/0021).

In the United Kingdom, most pregnant women receive care through the NHS. In the NHS, primary care, maternity care, and speciality teams operate separately, often in different organisations. In pregnancy, women typically have their first antenatal appointment with a midwife and, following a risk assessment, will be allocated to midwife‐led or consultant‐led maternity care with input from specialty teams where required.

### 2.1 Sampling and Recruitment

Purposive sampling of women was guided by public and professional stakeholders, and MuM‐PreDiCT studies which identified the most common or serious MLTCs in pregnancy, working closely with public contributors guided by the Improving Inclusion of Under‐Served Groups in Clinical Research (INCLUDE) and Increasing Participation of Black Asian and Minority Ethnic groups in Health and Social Care Research recommendations [21, 22]. Purposive sampling of healthcare professionals prioritised key groups, informed by women's interviews, and public and professional stakeholders. Snowball sampling was used for professional participants to identify others in their network. Multiple recruitment methods were used to maximise reach and diversity of the sample.

#### 2.1.1 Open Recruitment

Posters and flyers were shared via social media, third‐sector and professional organisations, and the researchers' networks. Interested individuals contacted researchers via the MuM‐PreDiCT website, email or telephone.

#### 2.1.2 NHS Site Recruitment

Five NHS trusts or health boards were purposively selected to provide diversity in case‐mix and service context (three England; one Scotland; one Northern Ireland). To recruit women, healthcare professionals identified women meeting eligibility criteria and provided study information. To recruit staff, a designated clinician at each site shared information by email and word‐of‐mouth. Potential participants either contacted researchers directly or consented to their details being shared.

Researchers sent a Participant Information Sheet and study reply slip to potential participants, which included details to confirm eligibility and facilitate sampling. For professionals this included location, job role, and role in the care of women with MLTC; and for women this included age, occupation, ethnicity and health conditions. Iterative assessment of the characteristics of existing participants guided subsequent efforts to recruit underrepresented groups, for example, targeted social media posts and site recruitment.

### 2.2 Data Collection

Before interviews, rapport was built with participants and researchers provided study information and an opportunity to ask questions. Participants had previously conversed with Author 1 via email or telephone during the study screening phase. Verbal consent was sought for telephone and online interviews and written consent for in‐person interviews. Between March 2022 and May 2023, interviews were conducted online (Microsoft Teams or Zoom), by telephone or in participants' homes by female Authors 1 and 2. Both authors were postdoctoral research fellows, experienced in conducting qualitative interviews. Interviews lasted approximately 45–60 min and, other than one interview where a woman attended with their partner, all interviews were conducted as one‐to‐one interviews. Reflective notes were taken following interviews, and no pilot or repeat interviews were conducted. Details on number of study refusals was not collected and no participants dropped out. Flexible interview guides and open‐ended questioning included important topics identified by our PPIE Advisory Group (Supporting Information S1). Women were asked questions pertaining to diagnosis, effects of their conditions on daily life and pregnancy, maternity care coordination, areas of their care that they were satisfied or not with, and future recommendations. Staff were asked questions pertaining to care organisation for women with MLTC, perceived effects of MLTC on women's experiences, impact of the COVID19 pandemic on care delivery, areas of care provision that work well and not, and future recommendations. Interviews were digitally audio recorded and transcribed verbatim. NVivo 12 was used to support data management and analysis [20]. Participants received a shopping voucher as compensation.

### 2.3 Analysis

Our qualitative analysis was interpretive. Data were analysed inductively, using thematic analysis [23]. Following familiarisation through listening to recordings and reading transcripts, Author 1 and Author 2 reviewed eight transcripts (four each) and independently generated initial codes. Authors S.J.H., S.M., L.L., and B.T. then discussed initial codes and developed a preliminary coding framework, which was applied to eight further transcripts and refined further. Author 1 and Author 2 iteratively developed codes into main themes and subthemes, supported by frequent discussion with L.L. and B.T. Theme development drew on academic and clinical knowledge among the research team and was informed by theory from the wider literature regarding care for single health conditions in pregnancy and for MLTC in other contexts. Through the analysis process, we drew on relevant theoretical lenses to support our understanding and interpretation of findings. Extracts are labelled using W for women and S for staff with the participant number.

While we did not share transcripts with study participants, reflections on the data analysis process were supported by discussion with the MuM‐PreDiCT lived experience PPI group. The PPI group included members with MLTC and recent experience of maternity care in all four countries of the United Kingdom. The group consisted of women from White, South Asian, and Black ethnic backgrounds residing in both rural and urban locations. Authors S.J.H. and S.M. met with the group online and shared anonymised interview transcript extracts with the group for their feedback and reflections. We also met with other members of the research team to further discuss and refine themes.

The research team considered their own backgrounds when analysing and discussing themes, specifically clinical roles and disciplinary backgrounds, personal experiences and stories they had previously heard. Each explicitly noted how these influences may shape their interpretations of data and invited team members to challenge and discuss these.

## 3 Results

### 3.1 Participants

Semistructured interviews were conducted with 57 women living with MLTC who were at least 28 weeks pregnant or had given birth in the last 2 years, and 51 NHS healthcare professionals with experience of caring for women with MLTC.

Participant characteristics are presented in Table 1 (women) and Table 2 (healthcare professionals). The sample represented 58 physical health conditions and 15 mental health conditions and mood disorders and consisted of women living in all four countries of the United Kingdom from diverse ethnic backgrounds. Professionals were working in 24 different roles.

**Table 1:** Women characteristics.

Characteristics	Frequency
Country of residence	
England	37
Scotland	12
Northern Ireland	4
Wales	4
Urban/Rural	
Urban	43
Rural	14
Age (years)	
18–25	5
26–30	13
31–35	22
36–39	16
≥40	1
Ethnicity	
White British/Welsh/Scottish/Irish	39
Mixed	3
Pakistani	3
British Asian	1
Malaysian	1
Indian Arab	1
Black British	2
Black African	1
Black Caribbean	1
Nigerian British	1
White South African	1
White Polish	1
White (other)	1
Romanian	1
Mental health & neurodevelopmental conditions	
Anxiety disorders	18
Depression	13
PTSD/C‐PTSD	7
Personality disorder	3
Psychosis	1
Bipolar	1
OCD	1
Anorexia nervosa	1
Dyspraxia	2
ADHD	2
Autism	1
Learning disability	1
Trauma dyslexia	1
Physical health conditions	
Neurological conditions (e.g., epilepsy, multiple sclerosis)	14
Rheumatic conditions (e.g., arthritis, lupus)	11
Gastrointestinal disorders (e.g., ulcerative colitis)	9
Cardiac/Cardiovascular	10
Gynaecological conditions (e.g., endometriosis)	6
Endocrine (e.g., diabetes)	6
Respiratory (e.g., asthma)	6
Obesity	4
Blood disorders (e.g., venous thromboembolism)	3
Cancer	1
Skin disorder	1
Renal (kidney) disorder	1
Deafness	1
Blindness	1
Other	4
No. of conditions	
2 conditions	27
3 conditions	13
4 conditions	9
5+ conditions	8
Combinations of health conditions	
Physical health conditions only	25
Mental health/Neurodevelopmental conditions only	3
Physical + Mental health/Neurodevelopmental conditions	29

Abbreviations: ADHD, Attention deficit hyperactivity disorder; C‐PTSD, complex‐posttraumatic stress disorder; OCD, obsessive compulsive disorder; PTSD, posttraumatic stress disorder.

**Table 2:** Healthcare professional characteristics.

Characteristics	Frequency
Country	
England	39
Scotland	9
Northern Ireland	2
Wales	1
Specialty/Job role	
Midwives	
Community midwife	2
Maternal medicine consultant midwife	1
Epilepsy	1
Maternal medicine	1
Infant feeding	1
Perinatal mental health	1
Infectious diseases	1
High‐risk pregnancies	1
Public protection	1
Senior midwife coordinator & research midwife	1
Hospital midwife	1
Doctors working in maternity services	
Consultant obstetrician & gynaecologist	3
Consultant obstetrician	3
Consultant foetal & maternal medicine	3
Specialty trainee obstetrics	3
Consultant anaesthetist	2
Consultant neonatologist	1
Specialty trainee neonatologist	1
Consultant obstetric physician	1
Obstetric physician (+consultant cardiologist)	1
Other secondary care professionals	
Consultant psychiatrist	2
Consultant haematologist	1
Diabetes consultant	1
Advanced nurse practitioner (epilepsy)	1
Consultant neurologist	1
Community/Primary care professionals	
General practitioner	3
Health visitor/Infant feeding lead	1
Consultant public health	1

Five main themes were developed: (1) *It takes a team*; (2) *Care navigation*; (3) *Multiple identities & the whole person*; (4) *Boundaries of expertise*; and (5) *Informational continuity.*

### 3.2 It Takes a Team

Women and professionals described the importance of teamwork, where ‘the woman has a role, is actually a partner in the care’ (S32, neonatologist). Often women received fragmented, inconsistent care and communication with professionals working in silos, focusing on one element of women's care without a holistic view. In some cases, this led to women feeling fearful and mistrusting of their care teams.

The Haematologist is like C‐section is an absolute no, unless needs must. Um, whereas the Obstetricians are the ones that keep mentioning it… And I genuinely think it's because, because it's not the same Obstetrician every time I go there, they don't know me… It's annoying because I feel like with the health conditions that I have, if it's not possible to have the same person looking after my care, then they should at least read about me before I go in there.

(W40, Venous thromboembolic disease, raised body mass index (BMI), 29 weeks pregnant)

Conflicting medication advice was common and women described searching for information themselves. Healthcare professionals also described challenges in changing medication decisions and the associated impact on women's trust (which they acknowledged may have been addressed by improved preconception conversations).

I was always under the care of obstetrician consultant, it was just a bit tricky because they never really knew what to do with me in relation to my meds for my arthritis, and of course my rheumatologist didn't really know what to do with me until I was being pregnant with the next one.

(W26, Ankylosing spondylitis, fibromyalgia, 5 months postpartum)

It feels very hard to undo the conversation where someone is told it's dangerous, because somebody has planted the seed of doubt than it is to change… Someone said “yes, they can do it”, and then you say, “Maybe on balance you shouldn't.” I don't know, it's much harder to undo. As soon as you have given the woman a lack of confidence that the medication is safe for her baby it's really difficult.

(S23, Consultant obstetric physician)

Some women described needing to be in control of all aspects of their care due to a lack of communication between, and confidence in, their care providers. One woman reported disorganised care in early pregnancy, which improved following the involvement of a specialist.

It sort of felt like unless I saw a specialist no one really knew what to do with me, and I had to be very on it myself … it was almost like people treat me like I was this bomb that was about to like go off or something… The specialist consultant didn't get involved until a bit later in the pregnancy. Once she got involved it felt a lot more joined up and she kind of took the lead on everything.

(W21, Postural tachycardia syndrome, Ehlers Danlos syndrome, ulcerative colitis, 6 months postpartum)

A team‐based approach was more challenging when healthcare professionals were reluctant to share or relinquish clinical responsibility, impacting on a woman's relationship with care providers. One woman described her consultant's reaction to her transferring to a different hospital.

The consultant there wasn't really pleased about me going to [place], I think it was a little bit ego, he couldn't deal with it because it was a professor and he was a consultant, and he said several times, “You don't need to go all the way to [place],” and I was like, “Well I think if you'd lost a child at full term through no fault of your own, and you'd done everything, and you'd asked for everything, then you'd go there too.”

(W15, Paroxysmal atrial fibrillation, generalised anxiety, 17 months postpartum)

This perspective was echoed by specialist midwives who recognised that consultants can become protective of their own patients, creating barriers to joined‐up care.

I think it would be useful… having joint clinics between conditions, because often the consultants… it can get territorial sometimes, and it can be, “This is my patient”.

(S41 Specialist midwife, high‐risk pregnancies)

Some healthcare professionals did not recognise their value as part of a woman's care team. One participant described difficulties in involving all relevant colleagues.

I had an MDT [multidisciplinary team meeting] just today about another woman who's got inflammatory bowel disease, and she's at [hospital name], the gastroenterologist who has looked after her for years didn't even reply to any emails that had been sent, didn't reply to the MDT invitation, and actually has counted himself out of delivering her care, which is a huge shame… I need that gastroenterology department to recognise the need for an MDT, and their involvement in it. So, I suspect they don't recognise the importance of their input as yet.

(S23, Consultant obstetric physician)

### 3.3 Care Navigation

Women described many instances where they took on the role of care navigator, for example logging appointments, emailing and telephoning services. They perceived that they would have experienced worse care and outcomes if they had not performed this role. While some were willing to do this work, they recognised that they were fortunate to be able to advocate for themselves.

I would have to feedback what consultant said to the midwife, I'd have to feedback what the other consultant said to the other consultant, and it was all on me to manage my own care, which I'm fine with, but I think if I wasn't proactive in managing my own care, if it was somebody else that was maybe more shy, or didn't know as much about their conditions, or hadn't managed them for so long, would they have got the same level of care that I did? …Things could have easily slipped through the cracks.

(W17, Hypothyroidism, ulcerative colitis, 16 months postpartum)

Healthcare professionals also recognised the importance of someone to coordinate a woman's antenatal care to avoid bouncing women between care teams.

What you want to try and avoid is patient tennis, and say “Oh no it's… I'm going to bat that away.”

(S30, Consultant obstetrician‐ maternal medicine)

A designated coordinating professional can offer continuity and oversight of care, connect providers, pool expertise, and provide someone that a woman can trust. This role was described as missing in maternity care.

If there's a cancer MDT it's not the consultants who are responsible for having to generate the lists of patients all the time, there will be a specialist either administrator or specialist nurse who has the lists of patients and then will bring it to the meeting, will make sure these patients are being followed up…In the pregnancy teams it's us having to do all of that stuff as well, which is problematic, because you're not the best placed person to do it.

(S07, Consultant in fetal and maternal medicine)

Various individuals described their role as a care coordinator and its importance in providing oversight of a woman's care. For example, one obstetric physician provided this function where multiple specialities were involved.

I think as an obstetric physician you do a lot of the clinical stuff, but when women have lots of specialty input you also act as a project manager. So, I don't need to make all those specialist decisions myself, but I am able to facilitate the forum in which we ask the questions of the specialist audience, and all come together to make a plan.

(S23, Consultant obstetric physician)

Specialist midwives and obstetricians also described their role in organising multidisciplinary team (MDT) meetings, especially in cases where women were experiencing physical, mental, and social complexities, and it was necessary to link up different specialists to provide holistic care.

I very often would organise MDTs. I think the consultants would probably arrange the ones where it's two quite clear medical conditions, or whatever, but I would say that when these conditions are complicated by mental health, and engagement, and vulnerability, that I would probably take the lead … or not take the lead, but that I would think to get in touch with consultants and say, “Can we have a meeting about this? Because I think we're struggling here, we need to link up together and see how we're going to best manage care for this person”.

(S27, Specialist midwife in public protection)

### 3.4 Multiple Identities and the Whole Person

Women described how care did not always account for their different identities; as a pregnant person, as a new mother and as someone with long‐term conditions. Of the women we spoke to, many were satisfied with elements of the care for their health conditions, although it was rare to be completely satisfied overall.

The whole team of people who have been looking after me with my blood condition, they have been amazing from start to finish and honestly could not fault it. Anything like, any questions, any problems, even just needing a new prescription, they are constantly on standby, for anything that I may need.

(W40, Venous thromboembolic disease, raised BMI, 29 weeks pregnant)

However, a few women felt they were seen as a combination of individual health conditions rather than a pregnant person, with little or no consideration for how their conditions and the pregnancy would impact on them as a whole.

No one really spoke to me, asked me about who I am as a person, what my conditions are … um, so, with my hypertension and my bone disease I was quite concerned about carrying a baby, what the impact would be and how it would affect me.

(W28, Osteopenia, high blood pressure, 10 months postpartum)

This focus on women's conditions often meant that they missed out on person‐centred antenatal care, such as input from a midwife to provide feeding and birth plan support.

…the community midwives didn't really want to see me, but the hospital were only focused on a … really small part to me of being pregnant, and I was missing that actual antenatal care … it's not really a consultant's job to talk to me about … how the kind of breastfeeding would go, because I had a really difficult time with my first baby with feeding. I had these types of questions and I felt like well I can't really ask the diabetic consultant these questions because it's not really their area, and then I didn't really want to phone up the community midwife line because you just leave a message.

(W05, Type 1 diabetes, ulcerative proctitis, coeliac disease, 11 months postpartum)

Overall, women expressed feelings related to a loss or lack of control throughout their pregnancy, a sense of not being kept in the loop or treated as a partner in their care. One woman described a mixed pregnancy experience, whereby she was treated as an equal partner until she was admitted to hospital with high blood pressure, when she felt out of control and unsupported.

But since that moment I told you that I went in and they had to call an ambulance, I actually became a puppet. I'm a social worker by background so that's what I do, so I am not an ignorant person…I support people, I work in the community as well…so I know how it feels like to be vulnerable as well. So I never thought that being such a strong person as I am I am going to become so vulnerable I am going to lose control so quickly.

(W12, Rheumatoid arthritis, hypertension, 20 weeks postpartum)

Postnatally, many women spoke about a deterioration in their own care and a much greater focus on the baby.

Once you have a baby the focus completely shifts, it's like you're love‐bombed when you're pregnant, and then when the baby comes it just drops like a hot rock.

(W26, Ankylosing spondylitis, fibromyalgia, 5 months postpartum)

One participant, who also worked as a health visitor, described how the 6‐week routine postnatal check with her general practitioner was focused solely on the baby with no attention towards her health conditions or birth experience.

They checked baby, but nothing to do with… not me. So, for my experience as a health visitor, I know most mothers receive phone calls anyway, but I just thought maybe for me it will be different because of my health conditions and how much blood I had lost. But the fact that they didn't was quite poor.

(W10, Endometriosis, polycystic ovary syndrome, anaemia, 5 months postpartum)

Postnatal care was specifically likened to ‘falling off a conveyer belt’ by two healthcare professionals. One described a lack of postnatal care funding as an issue where the ‘job is done’ if the mother and baby survive, and another shared concerns about a lack of specialist postnatal follow up.

I guess the intensity through pregnancy is probably quite high, and then I think they fall off the conveyer belt at the end, baby is born…. You hope somebody picks them up, because the teams that look after them during pregnancy, the cardiac teams for example, don't always look after them post‐pregnancy. So, some of them will, but not all of them, because the gastro consultants, the cardiology consultants that manage them in the pregnancy have their own cohort of patients, they don't always manage that condition outside of pregnancy.

(S11, Consultant in Obstetrics & Gynaecology)

Linked to this, one general practitioner detailed challenges with arranging appropriate care after pregnancy.

Postnatally, depending on the timeframe they may then get that level of service again, which is great, but you could end up being in a really sticky situation as a GP, where you have someone who is maybe then, I don't know, eight weeks, three months postpartum, and it probably isn't a postpartum issue, probably isn't related to their pregnancy, but it's very relevant that they have been pregnant recently. So then they would for example be under cardiology, well the wait time for cardiology is about six/seven months at the moment, maybe longer, I don't know, and so then you end up then being quite stuck with these individuals, because you can't access that same level of care that they had before, and then I think for the patients as well that can feel like a little bit confusing as well as to why they have suddenly got this massive downgrade in service.

(S16, General Practitioner)

### 3.5 Boundaries of Expertise

Women appreciated honesty from healthcare professionals when they had reached their knowledge limits. In turn, this created a communication balance where women were listened to and treated as the expert in their conditions.

The honesty from the midwives and the nurses on the ward was refreshing … for them to go, “We don't know much about Crohn's, that isn't our area.” So I knew where I stood with them, and they knew where they stood with me with regards to medication and treating me more like the expert of my Crohn's. They're the expert of babies and all of that, but I'm the expert of my Crohn's, and I know when it's affecting me.

(W37, Crohn's disease, blood clots, 8 months postpartum)

Other women felt that their expertise was not recognised, and they were not taken seriously, to the extent that they started questioning their own embodied knowledge.

So I think it was a mix of not being listened to and ignorance on the part of some of the older more senior staff, just saw the wheelchair and basically refused to listen to what I was saying point blank, and literally every time I was trying to tell them they were like, “No, you're wrong, there's no way that you can't feel movement,” this, that and the other, and literally just trying to almost gaslight me and make me think, “Hang on a second, am I crazy?”

(W16, Ehlers‐Danlos syndrome, spondylolisthesis, severe asthma, functional neurological disorder, PTSD, 12 months postpartum)

Women's accounts demonstrated that they value midwifery input, but often midwives reported low confidence in managing women's health conditions, particularly if they had not trained as nurses before becoming midwives. This emphasises the importance of pooling expertise and skills and MDT working to provide comprehensive care.

I was direct entry [to midwifery training], so I wasn't even a nurse before, I feel like we weren't really trained on it at all. I remember we did have… in year two we did have some lectures, but it was mostly comorbidities, not multiple morbidities on top of pregnancy … that's where some people say it's quite handy to be a nurse beforehand, so they've got that knowledge and experience as well.

(S07, Midwife)

Further, women could be fearful both when they perceived health care professionals (HCP)s to be unjustifiably over‐confident in their assertions, and when they detected unacknowledged anxiety in the demeanour of HCPs about managing their conditions.

They were lovely, but as I said they [midwives] don't know much about the ins and outs. I feel like when I said I had bipolar disorder they were a bit like “Oh my god what's that? What do I need to do?” You could just see it on their face panic and think … it makes you feel like “Oh god I have got a really bad condition?” And it is a bad condition, but it makes you feel worse that you can see on their face that they are panicked by it.

(W14, Bipolar, anxiety, 16 months postpartum)

Healthcare professionals recognised that their concerns about complex care could generate anxiety among women. One participant suggested that some professionals avoided complex cases due to a lack of knowledge and experience.

…for colleagues that aren't as experienced, there's a real, at times, a real fear of looking after these groups of women, and a real anxiety, and that stems from lack of experience, lack of understanding, and it's kind of a running joke amongst specialists and physicians, that if there's a pregnant woman that's admitted with a problem, there's a real, “Oh, let's run away because this is getting scary and difficult,” and that transmits to the women. It does transmit to the women—because I see it in their eyes and I hear it from them, and it breaks my heart.”

(S38, Consultant obstetric physician)

Some professionals detailed the importance of acknowledging women's expertise without preconceptions.

Never undermining the woman's knowledge as well, because you need to do that assessment, because the woman may be extremely knowledgeable about her own condition, and whatever you must do you must never patronise that knowledge, and it's about building up on that knowledge wherever the woman is starting from.

(S03, Epilepsy specialist midwife)

### 3.6 Informational Continuity

Many women and professionals described difficulties accessing information as a barrier to joined‐up care, specifically the setup of information technology (IT) systems and variation in electronic patient records systems between clinical specialties and healthcare organisations.

A few of them were quite anti IT, because they just wanted paper notes, which again I understood, I never held against them, because I wanted paper notes too…. But it felt like with [electronic patient record] because the way they had to input stuff was all these dropdowns and prompts it just felt like half the information had gone, I don't know, into the ether. So, I have quite brain fog anyway, I have ever since I had ank spond, fibromyalgia, I've always been a bit foggy, being pregnant on top having just had COVID I was all over the shop. It was a bit, what did they say? “Oh, check [electronic] notes, oh that doesn't know either”.

(W26, Ankylosing spondylitis, fibromyalgia, 5 months postpartum)

So, our notes at the [hospital] are computerised on [electronic patient record], so whenever they come in to the [hospital] we can access those and we can see it straight away, and generally they will be planned for different scenarios… I guess what doesn't work well is when they present at other hospitals that have less experience, and they can't then see the plans that have been put in place, and that tends to be when we have the worst outcomes.

(S06, Specialist trainee (6th year) in fetal and maternal medicine)

Both women and staff described challenges in sharing timely information and coordinating care across multiple sites and organisations.

I felt like the co‐ordination of it wasn't very good at all, because I was speaking to the obstetrician and they said this is my consultant, he's in another hospital but is the same health board. But they couldn't get in touch, and I found it very frustrating, because I had to contact them to say, “Doctor [name]…” or whatever her name, “wanted to contact you, she needs to speak to you,” and it was so stressful. How hard is it for people … working in a same health board to get in touch? And then in the end I had to ask him to write her a letter so that he had her contact details.

(W42, Eosinophilic asthma, anxiety, depression, 2.5 years postpartum)

Healthcare professionals shared concerns about the identification of high‐risk women, and the reliance on women to communicate between teams, particularly in acute situations and when electronic records were not accessible to all specialties.

Where it's a really big problem and it's very frustrating is that some of your highest risk patients who are perhaps under a psychiatric team they're cared for by a psych team in another Trust, another hospital, another bit of town, and we don't have a way of seeing their records, and they don't have a way of seeing our records. So something really big could happen in that woman's pregnancy and they wouldn't know unless she tells them, which I think is a really bad way to rely on providing joint care.

(S16, Consultant psychiatrist)

## 4 Discussion

Study findings indicate that having two or more pre‐existing health conditions while pregnancy results in multiple, cumulative challenges for women. This can result in a substantial burden of responsibility for women, including the need to maintain communication with their care team, and coordinate their care. Many feel vulnerable, not listened to, and let down by a lack of acknowledgement of their expertise. Women and healthcare professionals highlighted fragmented care, poor communication and records access issues, and a lack of continuity and midwifery support. Inconsistent advice, particularly around medication was a challenge, with gaps in healthcare professional knowledge of health conditions. Two theoretical concepts can help to frame the findings: Responsibilisation and Burden of Treatment Theory. Responsibilisation has its roots in critiques of neoliberalism [24]. McGregor argues that neoliberalism is founded on three principles: ‘individualism, free markets via privatization and deregulation, and decentralization’ [25, p. 2]. In the context of healthcare, one perspective on this individualised, independent focus is that patients are being freed or ‘empowered’ to manage their care. It is reported that the more ‘activated’ the patient, the better their outcomes [26]. An alternative view is that governments and state organisations have passed responsibility back to individuals, allocating burden and potentially blame and downplaying wider structural determinants of health. Brown argues that ‘personal responsibility has been increasingly incorporated into welfare state policies, particularly since the 1970s [27]. There continues to be a growing trend for ‘responsibilisation’ (often related to ‘personalised healthcare’) which assumes that agents can (and should) be held morally responsible for their health outcomes’ (p. 695).

Responsibilisation has been a feature of the discourse around pregnancy [28, 29, 30, 31], a time of particular risk and in which parents (especially mothers) find themselves accountable for the health and safety of the unborn child. Lupton (2012) describes how ‘pregnant women are subject to imperatives which expect them to engage in an intense ascetic regime of self‐regulation and disciplining of their bodies’ [30, p. 329].

Burden of Treatment Theory is one expression of responsibilisation, examining the work delegated to individuals by the health system. In developing the theory, May et al. note that patients—especially those with MLTC—face ‘new and growing demands to organize and co‐ordinate their own care, to comply with complex treatment and self‐monitoring regimens, and to meet a whole range of expectations of personal motivation, expertise and self‐care’ [32, p. 2]. This is not simply the well‐documented burden of illness itself, but rather the proactive work involved in managing treatment. In contrast with the suggestion that patient activation can improve health outcomes, May et al suggest patients may struggle with ‘the array of tasks expected of them’ and that the weight and complexity of the burden could lead to noncompliance, and over‐ or under‐use of healthcare. They argue that likely consequences are poor outcomes for individuals and rising demand and costs of care.

This burden is complicated enough in the case of MLTC; but pregnancy creates a fresh set of tasks. People with MLTC may have evolved a way of juggling the work expected of them across different specialities, but pregnancy threatens to destabilise any fragile balance they have managed to establish, and the additional all‐consuming work may take away the enjoyment of pregnancy.

The 2022 MBBRACE‐UK national maternity surveillance enquiry into maternal deaths and morbidity presented new recommendations for the care of pregnant women with MLTC based on the review of select maternal mortality reports with a focus on diabetic ketoacidosis [6]. Although it was recommended that women may require multidisciplinary care from multiple teams in different locations [6], there is a lack of evidence outlining what multidisciplinary care should include or how it can most effectively be provided [33]. Existing guidance exists for adults with MLTC, but there is a need for improved implementation during pregnancy to provide maternity care which actively considers MLTC and ensures women know who is responsible for co‐ordinating their care [6]. Care coordination is a key principle of MLTC care [34]. A named co‐ordinator has been identified as important for women with both a physical and mental health condition receiving multidisciplinary care [33]. Our findings demonstrate that one of the chief burdens women with MLTC face in pregnancy is the work of coordination, and bridging the gaps in knowledge and conflicting advice which healthcare professionals readily acknowledge exist. The boundaries of expertise outlined above leave the woman standing alone, with no‐one to guide her. One strategy being used to overcome this is the development of Maternal Medicine Networks (in England). These networks provide clinical leadership and co‐ordinate specialist multidisciplinary care for women with significant medical problems, including those with MLTC [35].

The role of a midwife providing continuity and co‐ordination of care as part of multidisciplinary collaborative maternity care for women with medical complexity has been identified as an evidence gap requiring further exploration [36]. Our study highlights the need for this coordination to continue into the postnatal period, to ensure continuity of follow up of pregnancy‐related care, for MLTCs, and to provide interpartum care and preconception care for future pregnancies. The ChroPreg randomised controlled trial demonstrated a midwifery care intervention which included supporting women with coordination of care during pregnancy with long term conditions improved satisfaction with care (though it made no difference to the trial primary outcome, total days women spent in hospital) [37]. The midwives in ChroPreg had 3‐days of training for this role [38]. This would be a relatively low‐cost way to improve women's experiences, not least in giving them the midwifery contact that many of our participants lacked.

Poor interoperability between electronic patient records, even within the same hospital, meant that important information was not shared, resulting in anxiety, gaps in care, and women feeling responsible for informational continuity. This is a systemic problem across healthcare systems and has been identified as a key risk in maternal death enquiries [5], but is particularly acute in the context of MLTC where care is often multidisciplinary across settings [6].

Each of the care recommendations here and in previous work require a focused approach to implementation and testing, potentially in the form of a maternity care bundle for women with MLTC, which has been shown to improve outcomes in other healthcare contexts (e.g., the Sepsis 6 bundle developed by Daniels et al.) [39].

### 4.1 Strengths and Limitations

This study was planned, conducted, and analysed by a multidisciplinary team, including patient stakeholders. We included a diverse sample of 57 women, providing a broad perspective on experiences of UK MLTC maternity care. We interviewed both healthcare professionals in many roles spanning preconception, pregnancy, intrapartum, postnatal and interpregnancy care, and a high proportion of ethnic minority women.

Many of the women we interviewed described high levels of self‐advocacy. While participants were drawn from many different groups, despite extensive effort, we were unable to recruit any participants who required an interpreter. To address the significant and unacceptable inequalities in maternal and infant mortality and morbidity, understanding and improving the experiences and care of women in disadvantaged and minority groups is crucial. Given the additional risks of MLTC in pregnancy, and that Black and Asian women, and women living in the most deprived areas experience barriers to accessing maternity care and are at a much higher risk and are more likely to die around the time of pregnancy [5], future work is urgently required to understand the intersectional experiences of women in these groups with MLTC.

### 4.2 Conclusion

This study provides valuable insights into the maternity care experiences of women with MLTC and their healthcare providers, and highlights gaps in current care provision for a high‐risk population. Findings illustrate the importance of multidisciplinary care, where clinicians acknowledge their professional limits, women's expertise is valued, and they are treated as partners in their care. The care navigation role some women adopt is a substantial burden, which many will struggle to perform, potentially widening inequalities. Future work should explore strategies to include a named care coordinator for women with MLTC. There is an urgent need to improve information sharing between health care teams and organisations, to ensure consistent and timely care without key information being missed. Postnatal care and follow up are particularly important areas for improvement to ensure optimal outcomes and experiences for women and babies in the short and long term. The findings underpin ongoing work by the MuM‐PreDiCT Consortium to coproduce recommendations for components of care to improve experience and outcomes for women with MLTC which can be tested empirically.

## Author Contributions

**Stephanie J. Hanley:** conceptualization, investigation, writing–original draft, formal analysis, methodology, project administration. **Sharon McCann:** conceptualization, investigation, formal analysis, writing–original draft, methodology. **Siang Ing Lee:** writing–review and editing, conceptualization. **Zoe Vowles:** writing–review and editing, conceptualization. **Rachel Plachcinski:** conceptualization, writing–review and editing. **Krish Nirantharakumar:** conceptualization, funding acquisition, writing–review and editing, supervision. **Mairead Black:** conceptualization, writing–review and editing, supervision. **Louise Locock:** conceptualization, investigation, methodology, writing–original draft, supervision, formal analysis. **Beck Taylor:** conceptualization, investigation, writing–original draft, methodology, supervision, formal analysis.

## Ethics Statement

This work was ethically approved by the Wales Research Ethics Committee 7 (reference [20]/WA/0021).

## Consent

All participants provided informed consent before participating in this work.

## Conflicts of Interest

The authors declare no conflicts of interest.

## Supporting information

Supporting information.

## Data Availability

Research data are not shared.

## References

[1] S. I. Lee , A. Azcoaga‐Lorenzo , U. Agrawal , et al., “Epidemiology of Pre‐Existing Multimorbidity in Pregnant Women in the UK in 2018: A Population‐Based Cross‐sectional Study,” BMC Pregnancy and Childbirth 22, no. 1 (2022): 120, 10.1186/s12884-022-04442-3. 35148719 PMC8840793

[2] R. D'Arcy , M. Knight , and L. Mackillop , “A Retrospective Audit of the Socio‐Demographic Characteristics and Pregnancy Outcomes for All Women With Multiple Medical Problems Giving Birth at a Tertiary Hospital in the UK in 2016,” BJOG 126 (2019): 128.30239131

[3] L. K. Admon , T. N. A. Winkelman , M. Heisler , and V. K. Dalton , “Obstetric Outcomes and Delivery‐Related Health Care Utilization and Costs Among Pregnant Women With Multiple Chronic Conditions,” Preventing Chronic Disease 15, no. 2 (2018): 170397, 10.5888/pcd15.170397. PMC5814150 29420168

[4] M. Knight , K. Bunch , R. Patel , et al., *Lessons Learned to Inform Maternity Care From the UK and Ireland Confidential Enquiries into Maternal Deaths and Morbidity 2018–2020* (Oxford: National Perinatal Epidemiology Unit, University of Oxford, 2022).

[5] M. Knight , K. Bunch , A. Felker , et al., *Saving Lives, Improving Mothers' Care. Lessons Learned to Inform Maternity Care From the UK and Ireland Confidential Enquiries Into Maternal Deaths and Morbidity 2019–2021* (Oxford: National Perinatal Epidemiology Unit, University of Oxford, 2023).

[6] C. Frise , S. Russell , T. Kelly , et al., “Messages on Caring for Women With Multiple Morbidities,” in *Saving Lives, Improving Mothers' Care Core Report—Lessons Learned to Inform Maternity Care from the UK and Ireland Confidential Enquiries into Maternal Deaths and Morbidity 2018–20*, eds. M. Knight , K. Bunch , R. Patel , J. Shakespeare , R. Kotnis , S. Kenyon , and J. J. Kurinczuk , on behalf of MBRRACE‐UK (Oxford: National Perinatal Epidemiology Unit, University of Oxford, 2022), 34–44.

[7] “Pregnancy With Another Condition or Disability.” Healthtalk, https://healthtalk.org/pregnancy/pregnancy-with-another-condition-or-disability.

[8] S. Wilson , “‘When You Have Children, You're Obliged to Live’^1^: Motherhood, Chronic Illness and Biographical Disruption,” Sociology of Health & Illness 29, no. 4 (2007): 610–626, 10.1111/j.1467-9566.2007.01008.x. 17498171

[9] A. Weckesser and E. Denny , “Women Living With Epilepsy, Experiences of Pregnancy and Reproductive Health: A Review of the Literature,” Seizure 22, no. 2 (2013): 91–98, 10.1016/j.seizure.2012.11.001. 23182977

[10] S. F. Flocco , R. Caruso , S. Barello , T. Nania , S. Simeone , and F. Dellafiore , “Exploring the Lived Experiences of Pregnancy and Early Motherhood in Italian Women With Congenital Heart Disease: An Interpretative Phenomenological Analysis,” BMJ Open 10, no. 1 (2020): e034588, 10.1136/bmjopen-2019-034588. PMC7044861 31980511

[11] M. de Wolff , A. S. Ersbøll , H. Hegaard , et al., “Psychological Adaptation After Peripartum Cardiomyopathy: A Qualitative Study,” Midwifery 62 (2018): 52–60, 10.1016/j.midw.2018.03.012. 29655005

[12] H. Thomas , “Women's Postnatal Experience Following a Medically Complicated Pregnancy,” Health Care for Women International 25, no. 1 (2004): 76–87, 10.1080/07399330490253256. 14742111

[13] M. Stafford , A. Steventon , R. Thorlby , R. Fisher , C. Turton , and S. Deeny , Understanding the Health Care Needs of People With Multiple Health Conditions. 2018.

[14] K. Palmer , A. Marengoni , M. J. Forjaz , et al., “Multimorbidity Care Model: Recommendations From the Consensus Meeting of the Joint Action on Chronic Diseases and Promoting Healthy Ageing Across the Life Cycle (JA‐CHRODIS),” Health Policy 122, no. 1 (2018): 4–11, 10.1016/j.healthpol.2017.09.006. 28967492

[15] A. Peart , C. Barton , V. Lewis , and G. Russell , “A State‐of‐the‐Art Review of the Experience of Care Coordination Interventions for People Living With Multimorbidity,” Journal of Clinical Nursing 29, no. 9–10 (2020): 1445–1456, 10.1111/jocn.15206. 32043672

[16] V. Struckmann , F. R. M. Leijten , E. van Ginneken , et al., “Relevant Models and Elements of Integrated Care for Multi‐Morbidity: Results of a Scoping Review,” Health Policy 122, no. 1 (2018): 23–35, 10.1016/j.healthpol.2017.08.008. 29031933

[17] M. K. Hansen , J. Midtgaard , H. K. Hegaard , L. Broberg , and M. G. de Wolff , “Monitored But Not Sufficiently Guided—A Qualitative Descriptive Interview Study of Maternity Care Experiences and Needs in Women With Chronic Medical Conditions,” Midwifery 104 (2022): 103167, 10.1016/j.midw.2021.103167. 34763179

[18] L. Kelly , J. J. Kurinczuk , O. Rivero‐Arias , R. Fitzpatrick , E. Gibbons , and F. Alderdice , “Exploring the Use of Health and Wellbeing Measures During Pregnancy and the First Year Following Birth in Women Living With Pre‐Existing Long‐Term Conditions: Qualitative Interviews With Women and Healthcare Professionals,” BMC Health Services Research 21, no. 1 (2021): 597, 10.1186/s12913-021-06615-w. 34162368 PMC8223316

[19] A. Tong , P. Sainsbury , and J. Craig , “Consolidated Criteria for Reporting Qualitative Research (COREQ): A 32‐Item Checklist for Interviews and Focus Groups,” International Journal for Quality in Health Care 19, no. 6 (2007): 349–357, 10.1093/intqhc/mzm042. 17872937

[20] NVivo. “NVivo 14—Leading Qualitative Data Analysis Software with AI Solution,” *Lumivero*. Published ahead of print, 2017. https://lumivero.com/products/nvivo/.

[21] National Institute for Health and Care Research. “Improving Inclusion of Under‐Served Groups in Clinical Research: Guidance From INCLUDE Project.” (NIHR, 2020). https://www.nihr.ac.uk/documents/improvinginclusion-of-under-served-groups-inclinical-research-guidance-from-include-project/25435.

[22] A. Farooqi , R. Raghavan , A. Wilson , et al., “Toolkit for: Increasing Participation of Black Asian and Minority Ethnic Groups in Health and Social Care Research.” 2023. https://www.rcslt.org/learning/diversity-inclusion-and-anti-racism/evidence-research/. 10.1186/s12874-021-01489-2 PMC8758375 35026996

[23] V. Braun and V. Clarke , “Using Thematic Analysis in Psychology,” Qualitative Research in Psychology 3, no. 2 (2006): 77–101, 10.1191/1478088706qp063oa.

[24] J. Hutchison and J. Holdsworth , “What Choice? Risk and Responsibilisation in Cardiovascular Health policy,” Health: An Interdisciplinary Journal for the Social Study of Health, Illness and Medicine 25, no. 3 (2021): 288–305, 10.1177/1363459319886106. 31692388

[25] S. McGregor , “Neoliberalism and Health Care,” International Journal of Consumer Studies 25, no. 2 (2001): 82–89, 10.1111/j.1470-6431.2001.00183.x.

[26] J. H. Hibbard and J. Greene , “What the Evidence Shows About Patient Activation: Better Health Outcomes and Care Experiences; Fewer Data on Costs,” Health Affairs 32, no. 2 (2013): 207–214, 10.1377/hlthaff.2012.1061. 23381511

[27] R. C. H. Brown , “Moral Responsibility for (Un)Healthy Behaviour,” Journal of Medical Ethics 39, no. 11 (2013): 695–698, 10.1136/medethics-2012-100774. 23315854 PMC3812898

[28] K. Coxon , M. Scamell , and A. Alaszewski , “Risk, Pregnancy and Childbirth: What Do We Currently Know and What Do We Need to Know? An Editorial,” Health, Risk & Society 14, no. 6 (2012): 503–510, 10.1080/13698575.2012.709486.

[29] L. Hinton , A. Chisholm , B. Jakubowski , S. Greenfield , K. L. Tucker , R. J. McManus , and L. Locock , “‘You Probably Won't Notice Any Symptoms’: Blood Pressure in Pregnancy—Discourses of Contested Expertise in an Era of Self‐Care and Responsibilization,” Qualitative Health Research 31, no. 9 (2021): 1632–1644, 10.1177/10497323211003067. 34116606 PMC8438769

[30] D. Lupton , “‘Precious Cargo’: Foetal Subjects, Risk and Reproductive Citizenship,” Critical Public Health 22, no. 3 (2012): 329–340, 10.1080/09581596.2012.657612.

[31] E. Lee , J. Macvarish , and J. Bristow , “Risk, Health and Parenting Culture,” Health, Risk & Society 12, no. 4 (2010): 293–300, 10.1080/13698571003789732.

[32] C. R. May , D. T. Eton , K. Boehmer , et al., “Rethinking the Patient: Using Burden of Treatment Theory to Understand the Changing Dynamics of Illness,” BMC Health Services Research 14, no. 1 (2014): 281, 10.1186/1472-6963-14-281. 24969758 PMC4080515

[33] D. Bick , D. C. Ashworth , F. Mayer , C. Taylor , and L. Howard , “High Risk Pregnancy Due To Mental and Physical Co‐morbidity. The FIGO Continuous Textbook of Women's Medicine‐ Obstetrics Module,” *Global Library Women's Medicine* (2021). 10.3843/GLOWM.411883.

[34] NICE. *Multimorbidity: Clinical Assessment and Management*. (2016). https://www.nice.org.uk/guidance/ng56.

[35] National Health Service. *Maternal Medicine Network Service Specification*. 2021. https://view.officeapps.live.com/op/view.aspx?src=https%3A%2F%2Fwww.england.nhs.uk%2Fwp-content%2Fuploads%2F2021%2F10%2FB0709_Service-specification-for-maternal-medicine-networks-October-2021.docx&wdOrigin=BROWSELINK.

[36] F. Alderdice , R. Malouf , J. McLeish , N. Roberts , and R. Rowe . *What Evidence Is Available About the Use and Effectiveness of Collaborative Midwife Continuity of Care Models to Improve Pregnancy Outcomes for Women With Medical and Obstetric Complexity? A Scoping Review* (Oxford: NIHR PRU‐MNHC, National Perinatal Epidemiology Unit, University of Oxford, 2022).

[37] M. G. de Wolff , J. Midtgaard , M. Johansen , et al., “Effects of a Midwife‐Coordinated Maternity Care Intervention (ChroPreg) vs. Standard Care in Pregnant Women With Chronic Medical Conditions: Results From a Randomized Controlled Trial,” International Journal of Environmental Research and Public Health 18, no. 15 (2021): 7875, 10.3390/ijerph18157875. 34360168 PMC8345548

[38] M. G. de Wolff , M. Johansen , A. S. Ersbøll , et al., “Efficacy of a Midwife‐Coordinated, Individualized, and Specialized Maternity Care Intervention (ChroPreg) in Addition to Standard Care in Pregnant Women With Chronic Disease: Protocol for a Parallel Randomized Controlled Trial,” Trials 20, no. 1 (2019): 291, 10.1186/s13063-019-3405-5. 31138296 PMC6537398

[39] R. Daniels , T. Nutbeam , G. McNamara , and C. Galvin , “The Sepsis Six and the Severe Sepsis Resuscitation Bundle: A Prospective Observational Cohort Study,” Emergency Medicine Journal 28, no. 6 (2011): 507–512, 10.1136/emj.2010.095067. 21036796

