# Supplementary material for: Lost in the System: Responsibilisation and Burden for Women With Multiple Long‐Term Health Conditions During Pregnancy
Source: Health Expect. 2024 Jun 14;27(3):e14104. doi: 10.1111/hex.14104 (PMC11176589; doi:10.1111/hex.14104)
Supplement: Supplementary file 1 — Supporting information. [file HEX-27-e14104-s001.docx]

**Supplementary Information**

***MuM-PreDiCT consortium***

The Multimorbidity and Pregnancy: Determinants, Clusters, Consequences and Trajectories (MuM-PreDiCT) consortium aims to determine the burden of pre-existing MLTC in pregnant women using electronic health records (Work Package 1). Work package 2 is the qualitative work package and results are presented here. Work package 3 involves retrospective cohort studies using electronic health records and the development of a core outcome set for women with MLTCs using Delphi survey and consensus approaches. An understanding of signals for medication in pregnancy is being sought through the conduct of pharmacoepidemiological studies (Work package 4). Further determinants of MLTCs are also being identified using various prediction models (Work package 5).

***Recruitment strategy***

Recruitment to the study was over a 14-month period (March 2022-May 2023). It was important for us to capture the experiences of women and staff from a wide range of backgrounds and with certain characteristics/conditions which took time for us to achieve. For example, initially we opened recruitment to any woman with any two or more long-term health conditions, and later on we focused both our open and site-based recruitments to women with certain conditions to align with findings from a confidential enquiry report on maternal deaths and other epidemiology work within the MuM-PreDiCT project. Recruitment materials were adapted to reflect our set focuses (e.g., ‘two or more long-term conditions including a cardiac condition) and research midwives were asked to focus recruitment on potential participants with these conditions. This, in turn, led to us conducting a large number of interviews and recruitment was closed once we were satisfied with the diversity of our overall sample (and guided by data adequacy).

Whilst we attempted to recruit NHS sites in each of the four nations, we were unable to identify a site in Wales. However, we recognised that it was important to recruit individuals working and receiving maternity care in Wales, as such this was one of our specific open-recruitment targets.

***UK maternity care system for women with multiple long-term health conditions***

In the UK, most pregnant women will have their first antenatal appointment with a midwife. Based on a risk assessment, the midwife will make onward referrals to specialist obstetrician-led care for some pregnant women with pre-existing long-term conditions. Depending on a woman’s needs and health complexities, this may also require the involvement of specialist teams (e.g., perinatal mental health) and, or obstetric physicians. Other women may continue with midwifery-led maternity care.

Recognising the increasing importance of caring for medical conditions that pre-date or arise in pregnancy, in 2021, the NHS in England outlined the service specification for the Maternal Medicine Network, with an emphasis on a multidisciplinary team approach. A three-tiered care system was proposed based on the complexity of the medical problem(s) and local expertise: management by the local unit, shared care between the local unit and maternal medicine centre, and management by the maternal medicine centre.
